# Screening for canine coronavirus, canine influenza virus, and severe acute respiratory syndrome coronavirus 2 in dogs during the coronavirus disease-2019 pandemic

**DOI:** 10.14202/vetworld.2023.1772-1780

**Published:** 2023-09-13

**Authors:** Hércules Otacílio Santos, Eliane Macedo Sobrinho Santos, Hérica da Silva de Oliveira, Wagner Silva dos Santos, Arthur Almeida Tupy, Elber Gomes Souza, Rair Ramires, Ana Clara Orneles Luiz, Anna Christina de Almeida

**Affiliations:** 1 Campus Araçuaí, Federal Institute of Northern Minas Gerais, Araçuaí, Minas Gerais, Brazil; 2 Espaço PET Clínica Veterinária, Salinas, Minas Gerais, Brazil; 3 Clínica Veterinária e Pet Shop Neres e Souza, Salinas, Minas Gerais, Brazil; 4 Zecão pet shop clínica veterinária, Salinas, Minas Gerais, Brazil; 5 Prefeitura Municipal, Araçuaí, Minas Gerais, Brazil; 6 Institute of Agricultural Sciences, Federal University of Minas Gerais, Montes Claros, Minas Gerais, Brazil

**Keywords:** coronavirus disease-2019, georeferencing, rapid tests, zoonosis

## Abstract

**Background and Aim:**

Although most cases of coronavirus disease-2019 (COVID-19) are in humans, there is scientific evidence to suggest that the virus can also infect dogs and cats. This study investigated the circulation of severe acute respiratory syndrome coronavirus 2 (SARS-CoV-2), canine coronavirus (CCV), and canine influenza virus (CIV) in domiciled and/or stray dogs from different locations in the State of Minas Gerais, Brazil, during the COVID-19 pandemic.

**Materials and Methods:**

In total, 86 dogs living in homes, on the streets, or in shelters in the cities of Taiobeiras, Salinas, Araçuaí, and Almenara were randomly selected for this study. The COVID Ag Detect^®^ Self-Test was used to detect SARS-CoV-2. The ACCUVET CCV AG TEST – CANINE CORONAVIROSIS^®^ was used to detect CCV, whereas canine influenza was detected using the ACCUVET CIV AG TEST – INFLUENZA CANINA^®^. All collected data were mapped using QGIS 3.28.1 for spatial data analysis and the identification of disease distribution patterns. Descriptive analysis of the collected data, prevalence calculations, odds ratios (ORs), and 95% confidence intervals, when possible, was performed.

**Results:**

Of the 86 animals tested, only one dog tested positive for SARS-CoV-2 using the rapid test for viral antigen detection. No animals tested positive for CIV. Canine coronavirus was detected in almost half of the animals tested in Almenara. Severe acute respiratory syndrome-CoV-2 had a low prevalence (1.16%), versus 15.62% for CCV. Although the results were not significant, the age and breed of animals appeared to be associated with the occurrence of CCV. The results indicated that younger animals were 2.375-fold more likely to be infected. Likewise, purebred animals were more likely to contract the disease (OR = 1.944).

**Conclusion:**

The results indicate the need to maintain preventive measures against CCV, canine influenza, and SARS-CoV-2 in dogs. More studies are needed to better elucidate the panorama of these diseases in dogs, mainly in underdeveloped and developing countries.

## Introduction

In recent years, the contribution of companion animals to the transmission and epidemiology of coronavirus disease-2019 (COVID-19) has been the subject of extensive research [1–9]. Although the evidence in the literature is insufficient to verify the hypothesis that these animals are sources of infection for humans [8, 9], it is important to conduct studies that address the role of severe acute respiratory syndrome coronavirus 2 (SARS-CoV-2) in acute respiratory diseases in domestic animals to develop effective protocols for the prevention and treatment of respiratory diseases in these animals.

Scientific studies have illustrated that canine and feline species can contract COVID-19 [3–7]. These animals can also develop COVID-19-related clinical signs similar to those in humans, including upper respiratory tract symptoms, which carry a poor prognosis in the presence of comorbidities [5–7]. Although SARS-CoV-2 has received extensive attention during the COVID-19 pandemic, infection with other respiratory and/or gastrointestinal viruses in addition to SARS-CoV-2 has implications for health and animal welfare, wildlife conservation, biomedical research, and public health.

Coronaviruses (*Nidovirales*; *Coronaviridae*) can cause diseases that manifest differently in humans and animals. Following coronavirus infection, both humans and animals develop respiratory and gastrointestinal diseases. In addition to these diseases, animals also frequently exhibit neurological and hepatic signs, often causing significant economic impacts [10–12]. Although coronavirus infection generally manifests in mild symptoms in dogs, the infection can be fatal when comorbidities such as parvovirus infection are present [12–14].

Coronaviruses are classified into two subfamilies, namely, Letovirinae and Orthocoronavirinae, with the latter including four genera: *Alphacoronavirus*, *Betacoronavirus*, *Deltacoronavirus*, and *Gammacoronavirus*. The classic coronavirus (*Alphacoronavirus 1*) was first described in 1971 in Germany, and it is associated with feline coronaviruses and porcine transmissible gastroenteritis virus [15]. Canine respiratory coronavirus (*Betacoronavirus 1* – canine coronavirus [CCV]) was identified in the United Kingdom in 2003, and it is most closely related to bovine coronavirus [14–16].

Canine influenza, caused by canine influenza virus (CIV), is a respiratory disease that clinically manifests as coughing, sneezing, fever, and nasal discharge in dogs [17, 18]. CIVs can be classified into the subtypes H3N8 and H3N2, which arose from the interspecies transmission of equine and avian influenza viruses [19].

Therefore, it is emphasized that the spread of influenza viruses between species is a continuous threat to public health. Different CIV strains can infect dogs and cats, resulting in clinical signs of varying severity and virus excretion [19, 20], and coinfection can lead to the emergence of new viral variants [19, 21]. In this context, it is important to investigate the occurrence of influenza in companion animals because they can become reservoirs and disseminators of influenza virus among family members, potentially threatening public health [19].

This study investigated the presence of SARS-CoV-2, CCV, and CIV in stray and/or domiciled dogs from different locations in Minas Gerais, Brazil.

## Materials and Methods

### Ethical approval

This study was approved by the Committee on Ethics in the Use of Animals of the Instituto Federal do Norte de Minas Gerais according to the consolidated opinion regarding the project protocol 12/2020.

### Study period and location

The study was conducted in the study was conducted from January 2022 to January 2023 in the municipalities of Taiobeiras, Salinas, Araçuaí, and Almenara in the state of Minas Gerais, Brazil.

### Animals and body samples

**Figure-1:**
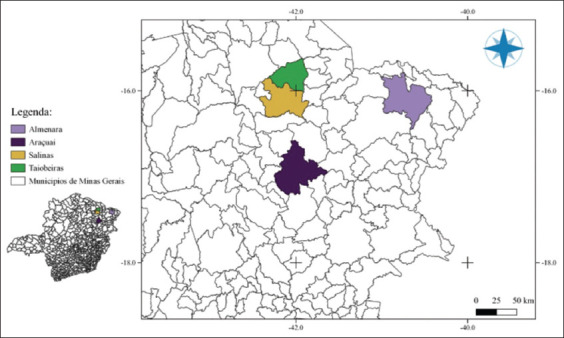
Representative map of the location of the study area [Source: QGIS 3.28.1].

The estimated population of dogs in the State of Minas Gerais ranges 2,141,192–3,425,908 (Minas Gerais, 2023). No statistical formula was used to determine the sample size and sampling was random and opportunistic. Dogs (n = 86) from households, streets, and shelters in Minas Gerais, Brazil, were included in this study (Figure-1). A brief characterization of the animals is presented in Table-1.

**Table-1:** Descriptive analysis of animals by location.

Location (MG)	Total amount	Housing		Sex		Race		Average age (Years)	Size		
			
		Wanderers	Domiciled	Male	Female	DR	SRD		S	M	L
Araçuaí	15	15	0	9	6	0	15	3.1	2	10	3
Taiobeiras	22	0	22	7	15	17	5	3.2	5	10	7
Almenara	21	1	20	11	10	13	8	6	14	5	2
Salinas	28	28	0	14	14	0	28	IND	9	10	9
Total	86	44	42	41	45	30	56	-	30	35	21

DR=Defined breed, SRD=No breed defined, IND=Undetermined [Source: QGIS 3.28.1].

All animals were photographed using a smartphone camera. The following data were collected and recorded: Breed, name (if any), sex, age (approximate), coat color, size (S, M, G), temperament, date and location of capture (if it is a shelter animal), and clinical signs (if present). The size of the animal was registered according to the American Kennel Club (AKC) guidelines [22]. According to AKC data, the average height and weight of a size S dog are 33.5 cm and 8.5 kg, respectively. The values for a size M dog are 53 cm and 22 kg, respectively, and those for a size G dog are 65 cm and 47 kg, respectively.

An appropriate muzzle was applied to each dog, which was physically restrained for the clinical examination. The clinical examination consisted of the following procedures: General observation and palpation of the body (good, fair, or poor state of health), assessment of the degree of hydration, evaluation of the skin and mucous membranes (oral, conjunctiva, genital, and anal), palpation of lymph nodes (mandibular, parotid, axillary, and popliteal) and palpable internal organs, cardiopulmonary auscultation, and rectal temperature measurement.

Nasal samples were collected to detect SARS-CoV-2 and CIV antigens. Rectal samples were collected to detect CCV. Samples were tested immediately after collection.

### Laboratory tests

All assays used in the study were commercially procured test kits according to the manufacturer’s instructions and analyses were conducted in the municipalities of Araçuaí, Taiobeiras, Salinas, and Almenara in Minas Gerais.

### Severe acute respiratory syndrome coronavirus 2

The COVID Ag Detect^®^ Self-Test (Batch: C055472, Validity: 01/30/2024), purchased from Eco Diagnóstica (Minas Gerais, Brazil, registered with ANVISA under No. 80954880183), was used to detect the SARS-CoV-2 antigen (Table-2). The COVID Ag Detect Autotest has a nitrocellulose membrane with two precoated lines: the control (C) and test lines (T). The control and test lines in the reading window are not visible before the sample application. The test line was precoated with anti-SARS-CoV-2 mouse monoclonal antibodies and the control line was precoated with anti-chicken immunoglobulin mouse monoclonal antibodies. Anti-SARS-CoV-2 mouse monoclonal antibodies were used to detect the SARS-CoV-2 antigen. During the test, the SARS-CoV-2 antigens present in the sample react with the anti-SARS-CoV-2 monoclonal antibodies conjugated to the colored particles, forming an antigen–antibody complex with the colored particle. This complex migrates through the membrane through capillary action to the test line, which was captured by anti-SARS-CoV-2 mouse monoclonal antibodies. A colored line will be visible in the result window if the SARS-CoV-2 antigen is present in the sample. If the antigen is absent in the sample, no colored line will appear in the test line. The control line is a procedural control and it should always appear if the test procedure was performed correctly and the control line test reagents were working.

**Table-2:** Information about the COVID Ag Detect^®^ Self-Test.

Parameters	Information
Methodology	Immunochromatography
Sample type	Nasal swab
Sample volume	4 drops
Test time	15 min
Sensitivity	96.38% (patients with symptoms) and 96% (asymptomatic patients)
Specificity	> 99%
Storage	2–30°C

Source: COVID Ag Detect^®^ Self-test Instruction Sheet

### Canine coronavirus

The ACCUVET CCV AG TEST – CORONAVIROSE CANINA^®^ (Hangzhou Biotech Co., Ltd.: V21090014, Val: 09/05/2023, China), an immunochromatographic assay, was used for the qualitative and differential detection of the CCV antigen in feces through rectal swabs. It has a sensitivity and specificity of 98.5% and 98.3%, respectively, for dog fecal samples.

In breif, the sample collected with the rectal swab was inserted into the diluent buffer tube. Three drops of the supernatant containing the sample and diluent were added to the “S” hole of the cassette, and the results were interpreted after 10 min. A colored line in the left part of the result window (C) indicates that the test is working properly. The right part of the result window (T) indicates the test result. The presence of a colored line indicates a positive result.

### Canine influenza

**Figure-2:**
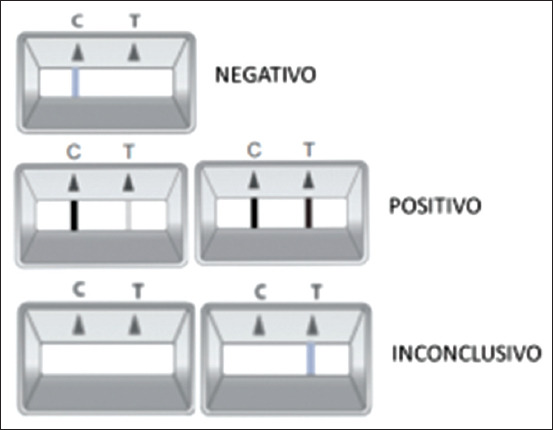
Scheme demonstrating the possibilities of results of rapid tests for the detection of antigens. The tests used are presented in the format of a plastic cassette containing the letters “C” and “T,” as control and test lines, respectively, on its surface. Lines are not visible in result windows before applying samples. The “C” lines are used for procedural control. These lines should always appear if the test procedure is correct and the reagents in the lines are working. A purple line will be visible in the “T” result window if the investigated antigens are present in the tested sample. Selected antibodies are used on the test band as capture and detection materials. Source: Package insert for rapid tests for antigen detection.

To detect CIV, we used the ACCUVET CIV AG TEST – INFLUENZA CANINA^®^ (Hangzhou Biotech Co., Ltd., China: V21090016, Val: 05/09/2023). This test is an immunochromatographic assay for the qualitative detection of the CIV antigen in ocular and/or nasal secretion samples for *in vitro* animal diagnosis. To perform the analysis, we collected nasal secretions using a swab, which was inserted into the sample diluent buffer. After shaking the swab in the buffer, four drops (100 μL) of the diluted sample were slowly added to the “S” hole of the cassette, and the results were interpreted after 10 min. A colored line in the left part of the result window (C) indicates that the test is working properly. The right part of the result window (T) indicates the test result. The presence of a colored line indicates a positive result. This test has a sensitivity and specificity of 97.20% and 95.30%, respectively (Figure-2).

### Georeferencing

All collected data was mapped using the QGIS 3.28.1 program (http://qgis.osgeo.org) for spatial data analysis and the identification of disease distribution patterns. The program was used to produce the COVID-19, CCV, and CIV prevalence maps and the location map of the municipalities studied within Minas Gerais. Using QGIS 3.28.1, it was possible to build and manipulate a structured geographic database through which the map was obtained. The coordinate reference system used was SIRGAS (Geocentric Reference System for the Americas) 2000, in UTM projection system, using the 24S zone.

### Statistical analysis

Statistical package for the social sciences^®^ version 23 (IBM SPSS Statistics for Windows, Armonk, NY, USA). was used for statistical analysis. A descriptive analysis of the collected data was performed to determine the relative and absolute frequencies of COVID-19, CCV, and canine influenza.

The prevalence of COVID-19, CCV, and canine influenza was calculated as the number of dogs that were positive for SARS-CoV-2, CCV, and CIV divided by the total number of dogs that were tested per municipality. Overall prevalence estimates were calculated for all dogs in the study.

For factors such as age, sex, breed, size, housing, and symptomatology in infected animals, odds ratios (ORs) and 95% confidence intervals were calculated. The factor difference between positive and negative animals was calculated using Fisher’s exact test. Final values were considered significant at p ≤ 0.05.

## Results and Discussion

Several reports have described dogs and cats infected with SARS-CoV-2 diagnosed through reliable tests such as reverse transcription-quantitative polymerase chain reaction (RT-qPCR) and anatomopathological investigations through necropsies [23, 24]. Of the 86 animals tested, only one dog (1.16%) had a positive result for SARS-CoV-2 in the rapid test that detects the viral antigen (Table-3). Although the rapid test used in this study was not produced for use in canine species, it is a specific test for the SARS-CoV-2 antigen. Therefore, the results are preliminary and further investigation is needed to determine if waves of COVID-19 occur again in the future. A recent study in a rural indigenous community in the Ecuadorian Amazon in South Amazon detected dogs, specifically stray dogs, infected by SARS-CoV-2. In this case study, three dogs tested positive for at least two SARS-CoV-2 viral targets [25]. SARS-CoV-2 investigations in companion animals in Latin America (Chile and Mexico) have also been reported in the literature [26, 27]. In another study, three cats tested positive for SARS-CoV-2 [28]. However, of 130 samples from companion animals tested by RT-qPCR for SARS-CoV-2, none tested positive for the novel coronavirus [27].

**Table-3:** Presence and geographic distribution of SARS-CoV-2 antigens.

Municipality of Minas Gerais	Number (%) of dogs tested	Number (%) of dogs positive for COVID-19
Araçuaí	15	0
Taiobeiras	22	0
Almenara	21	0
Salinas	28	1 (3.57%)
Total	86	1 (1.16%)

It is important to emphasize that the tests on animals were conducted outside the COVID-19 waves in Brazil and during a period when vaccination against the disease was extremely advanced in the human population. It is uncertain if the number of animals that tested positive would have been higher because study been conducted at the height of the epidemic in the country. In other studies that did not detect the presence of SARS-CoV-2 in companion animals of COVID-19-positive owners, the samples were collected from the animals long after the owners had a positive diagnosis [28–30].

Meanwhile, in a study in which animal samples were collected during the COVID-19 outbreak in humans, some animals tested positive for SARS-CoV-2 [25]. In other studies, the duration between the diagnosis of COVID-19 in humans and the collection of animal samples to investigate the presence of SARS-CoV-2 was not reported [31–35].

**Figure-3:**
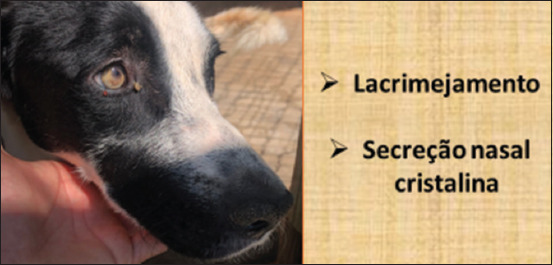
Photo of the dog that tested positive for COVID-19 by the COVID Ag Detect^®^ rapid test.

The animal that tested positive for SARS-CoV-2 lived in a municipal shelter for abandoned dogs in Salinas. At the time of the test, the animal had nasal secretions and eye tearing with no evidence of further respiratory signs (Figure-3). Notably, this animal had close contact with humans and other animals in the shelter.

It was not clarified whether individuals who maintained daily contact with the animal had COVID-19 around the time of testing in the animal. Therefore, it is impossible to prove the association with super-dissemination events [36]. Because of the lack of information, dismissing the possibility that the animal contracted the virus through close contact with workers and/or visitors at the shelter is impossible. Although this study and prior research obtained no evidence of the potential of domestic dogs to spread SARS-CoV-2 [28–30], it is important to perform more studies in underdeveloped and developing countries, particularly studies targeting abandoned animals, which are more vulnerable to infections.

Zambrano-Mila *et al*. [25] argued that anthropogenic transmission of SARS-CoV-2 is the most plausible explanation for the infection of stray dogs. This hypothesis is supported by the results of research conducted by these authors. In a study in which all dogs tested positive for two or more targets of the SARS-CoV-2 gene, the authors observed, in addition to a high infection rate in the human population studied (~90%), the presence of individuals with high viral loads.

No positive results were observed among the animals tested for CIV (Table-4). To clarify the importance of dogs and cats in the epidemiological chain of influenza virus, studies involving these animals are ongoing in several countries globally [37]. Giese *et al*. [20] reported that although dogs developed a mild respiratory disease, the potential role of these animals in the adaptation process of the highly pathogenic avian subtype to domestic mammals and its consequent transmission to humans should be considered.

**Table-4:** Detection of canine Influenza virus antigens.

Municipality of Minas Gerais	Number of dogs tested	Number of dogs positive for Canine Influenza
Taiobeiras	22	0

Canine influenza virus has been considered endemic or native to New York, New Jersey, Florida, and the Colorado-Wyoming border area [38], and it can be transmitted rapidly between individual dogs. In addition, higher mortality rates have been observed in young, old, and debilitated animals [39].

Although CIV has high morbidity, its mortality is low [40]. To date, two different CIV subtypes have been identified, namely, H3N8 and H3N2. The transmission of these viruses usually occurs through droplets expelled from the mouth or eyes of animals by sneezing or coughing [41]. Therefore, surface hygiene measures, population density control in shelters, and monitoring of infected animals are essential to contain viral dissemination. Although CIV was not detected in the sampled dogs in this study, it is important to maintain vigilance because of the dissemination and infectivity of the virus.

Canine coronavirus was detected in almost half of the animals tested in Almenara (Table-5). This virus is globally distributed and is especially prevalent in Europe [42] and Asia [43]. In Brazil, Pinto [44] conducted a study that identified CCV in the canine population. The identification of CCV and its variants is important for monitoring its spread in the Brazilian canine population, thereby contributing to the study of its phylogenies and epidemiology.

**Table-5:** Presence and geographic distribution of coronavirus antigens.

Municipality of Minas Gerais	Number of dogs tested	Number (%) of dogs positive for Canine Coronavirus
Araçuaí	15	0 (0%)
Almenara	21	10 (47.61%)
Salinas	28	0 (0%)
Total	64	10 (15.62%)

Although most dogs were negative for CCV, monitoring and investigation of the disease must be constant to prevent its spread. Canine coronavirus is shed in the feces for up to 2-week post-infection; this period is sometimes extended up to 180 days. Healthy dogs can excrete the virus in their feces for long periods [45].

**Figure-4:**
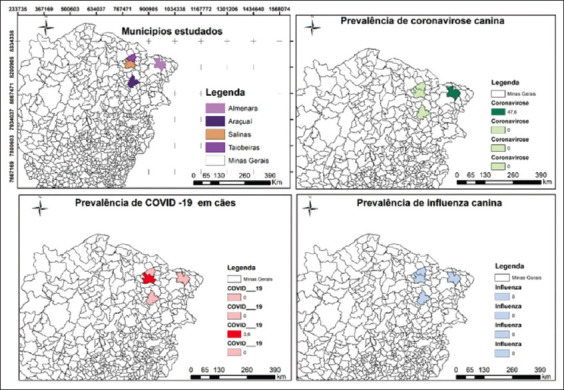
Situation of the occurrence of the diseases COVID-19, Influenza, and Coronavirus in dogs in four municipalities in the state of Minas Gerais, Brazil [QGIS 3.28.1].

This study estimated the prevalence of viral agents in dogs in the cities of Taiobeiras, Salinas, Araçuaí, and Almenara. Although no animals tested positive for CIV and SARS-CoV-2 had a low prevalence (1.16%), the prevalence of CCV was much higher (15.62%, Table-6 and Figure-4).

**Table-6:** Combined prevalence of SARS-CoV-2, Influenza virus, and canine Coronavirus antigens.

Analyzes	Araçuaí		Taiobeiras		Almenara		Salinas	
			
	SARS-CoV-2	Canine coronavirus	SARS-CoV-2	Canine influenza virus	SARS-CoV-2	Canine coronavirus	SARS-CoV-2	Canine coronavirus
Number of positive dogs/Number of dogs tested	0/15	0/15	0/22	0/22	0/21	10/21	1/28	0/28
Percentage of dogs tested that were positive (95% CI)	NA	NA	NA	NA	NA	47.6	3.6	NA

ND=Not determined, CI=Confidence interval

The hemagglutination inhibition test has already proved the circulation of the influenza virus among dogs in Brazil. However, the animals were seropositive for human influenza A viruses H1N1 and H3N2 and equine influenza A viruses H7N7 and H3N8 [46].

The prevalence of CCV infection has been examined in several countries globally, including Brazil. Studies in Japan, Italy, and Turkey recorded a serological prevalence of coronavirus in dogs with diarrhea of 44.1%–74.3% [47–49]. In Brazil, Mosca [50], Guirão [51], and Dezengrini *et al*. [52] estimated prevalences of 68.8%, 47%, and 50.4%, respectively. It should be noted that the prevalence is significantly higher in places with higher population densities than in individually housed dogs [53].

Corroborating the data of this study, other authors have identified a low prevalence of COVID-19 among pets in Brazil and in other countries [54]. Stevanovic *et al*. [55] reported that seropositivity among companion animals in Croatia is low, especially when compared to the results from China.

The risk factors for CCV in Almenara were studied. The evaluated factors (Table-7) were observed in both the positive and negative groups. Although there was no statistical difference between the groups, the age and breed of animals were potentially associated with the occurrence of CCV infection. These results indicate that younger animals are 2.375-fold more likely to be infected. Likewise, purebred animals were more likely to contract the disease (OR = 1.944). Dogs of all ages and breeds are susceptible to CCV infection. However, puppies are more susceptible, often develop clinical signs of enteritis, and have higher mortality rates. The disease occurs more frequently in kennels, shelters, and places where dogs live together. The virus is highly contagious and spreads rapidly in the canine population [56].

**Table-7:** Risk factors associated with the positivity of canine coronavirus for 21 dogs tested in the municipality of Almenara (Minas Gerais).

Variables	Number of positive dogs (%)	Number of negative dogs (%)	OR	95% CI	p-value
Age					
≤1 year	20	0	2.375	1.402–4.024	0.214
>1 year	80	100			
Sex					
Male	50	54	0.833	0.150–4.636	0.590
Female	50	46			
Race					
Pure	70	54	1.944	0.322–11.756	0.392
Mestizo	30	46			
Size					
Small	70	64	*	*	0.926
Average	20	27			
Big	10	9			
Housing					
Domiciled	90	100	0.450	0.277–0.731	0.476
Wandering	10	0			
Symptomatic					
Yes	80	82	0.889	0.101–7.856	0.669
No	20	18			

* Estimated risk cannot be calculated. OR=Odds ratio, CI=Confidence interval

## Conclusion

Canine influenza virus was not detected in the animals sampled in this study. Severe acute respiratory syndrome-CoV-2 was present in an insignificant number of animals, whereas CCV was more prevalent. More research is needed to clarify the role of companion animals in transmitting SARS-CoV-2 to other companion and wild animals and humans, especially in low- and middle-income countries where infection is frequent in stray and wild dogs and cats in rural and urban areas. This is because SARS-CoV-2 has a higher prevalence in humans, but several species of animals are susceptible to the virus. It is concluded that there is no justifiable reason to regard dogs as sources of SARS-CoV-2 in the Brazilian State of stray and wild dogs and cats. Observation of all preventive and sanitary measures to reduce the occurrence of canine influenza, CCV, and COVID-19 in animals is highly recommended [57, 58].

## Authors’ Contributions

HOS and EMSS: Study design and conception, drafted the manuscript and data analysis. HSO: Literature review and manuscript formatting. WSS: Literature review and manuscript formatting. AAT, EGS, RR, and ACOL: Collected the samples and data. ACA: Drafted the manuscript and data anal­ysis. All authors have read, reviewed, and approved the final manuscript.
